# Shoulder pain and functionality in Type-2 diabetic patients: A cross-sectional study of relationships with HbA1c levels and diabetes complications

**DOI:** 10.12669/pjms.41.8.12122

**Published:** 2025-08

**Authors:** Egemen Tural, Akın Dayan

**Affiliations:** 1 Egemen Tural Specialist, Family Medicine, Cifteler Family Health Center, Eskisehir, Turkey.; 2 Akın Dayan Associate Professor, Family Medicine, Department of Family Medicine, Haydarpaşa Numune Training and Research Hospital, Istanbul, Turkey

**Keywords:** Diabetes Mellitus Type-2, Diagnosis, Shoulder, Shoulder Pain

## Abstract

**Objective:**

The prevalence of shoulder pathologies in diabetics is higher compared to non-diabetics. However, the exact reasons for this increased incidence remain unclear. This study aimed to assess shoulder pain and function in type two diabetes patients and explore their relationship with HbA1c levels and microvascular complications.

**Methods:**

Our prospective cross-sectional study involved 184 type two diabetic patients seen at the Family Medicine Diabetes Polyclinic of the Training and Research Hospital between July 18, 2022 to October 18, 2022. Patients provided sociodemographic data, diabetes duration, and were assessed using the UCLA Shoulder Score. Medical records supplied information on microvascular complications and recent HbA1c levels.

**Results:**

Among the study cohort, 52.7% (n=97) were female. The median age was 59.5 years, with a median HbA1c level of 7.4% and a median diabetes duration of 14.5 years. Female participants demonstrated significantly lower UCLA Shoulder Scores compared to males, whereas higher educational attainment and income levels were positively associated with improved scores. Furthermore, there were significant inverse correlations between UCLA Shoulder Scores and diabetes duration, age, and body mass index. Patients presenting with diabetic retinopathy, nocturnal shoulder pain, or restricted shoulder mobility exhibited markedly reduced UCLA Shoulder Scores.

**Conclusions:**

In this study, 34.2% of patients with Type-2 diabetes demonstrated poor shoulder function as measured by the UCLA Shoulder Score. The findings emphasize the importance of routine shoulder assessments in diabetic patients, particularly those with retinopathy, obesity, prolonged disease duration, female sex, or low socioeconomic status. Enhancing physician awareness of musculoskeletal complications in diabetes is essential. Future multicenter studies and a multisystemic approach incorporating musculoskeletal evaluation into diabetes management are recommended to improve understanding and care of shoulder pathologies in this population.

## INTRODUCTION

Musculoskeletal (MS) disorders are more common in individuals with Diabetes Mellitus (DM). MS disorders include adhesive capsulitis (AC), carpal tunnel syndrome, stenosing tenosynovitis, Dupuytren’s contracture, limited joint mobility, diabetic neuroarthropathy, diabetic muscle infarction, calcific periarthritis of the shoulder, and diffuse idiopathic skeletal hyperostosis.^1^ Two of the most common shoulder pathologies are AC, also known as frozen shoulder, and rotator cuff (RC) pathologies.^2^ Studies suggest that 30% to 80% of individuals with Type-2 Diabetes Mellitus (T2DM) may experience musculoskeletal complications.^3^ Musculoskeletal disorders, despite their increased prevalence and significant social impact, are less extensively studied compared to other diabetic complications.^4^

Studies have demonstrated a higher prevalence of shoulder symptoms in diabetic patients (11% to 35%) compared to non-diabetic controls (2% to 17%).^5^ Out of all symptoms, shoulder pain emerges as the most frequent, marked by discomfort and limited mobility.^6^ This pain can impede daily activities, potentially impacting the body’s metabolic processes and overall quality of life.^7^

The underlying mechanisms behind the increased prevalence of shoulder pathologies in individuals with diabetes remain unclear.^8^ Current evidence suggests two main pathophysiological pathways: one involving connective tissue degeneration in the RC and joint capsule, and the other linked to peripheral or autonomic neuropathy. Recent studies have indicated that the accumulation of advanced glycation end-products (AGEs) due to chronic hyperglycemia impairs collagen structure and compromises connective tissue integrity.^9^,^10^

Moreover, AGEs are implicated not only in musculoskeletal damage but also in peripheral nerve injury and vascular complications associated with diabetes.^11^,^12^ A recent review highlights the potential role of fasting glucose and HbA1c in these processes, but emphasizes the need for further research to elucidate the complex interplay between metabolic disturbances and shoulder dysfunction in diabetic patients.^13^ The aim of this study was to evaluate shoulder pain and functionality among patients diagnosed with T2DM. Furthermore, the study seeks to explore the relationships between shoulder pain, impaired functionality, HbA1c levels, and diabetes-related microvascular complications in this patient population.

## METHODS

A prospective cross-sectional study was conducted among T2DM patients who presented to the diabetes clinic of the family medicine department at the training and research between July 18, 2022 and October 18, 2022.

### Ethical Approval:

The study was approved by the Clinical Research Ethics Committee of Haydarpaşa Numune Training and Research Hospital, University of Health Sciences, with the decision number HNEAH-KAEK 2022/131-3708; dated: June 13, 2022.

Upon reviewing hospital records, it was found that an average of one hundred patients per month and a total of 300 patients over three months visit the Family Medicine Diabetes Clinic. The population of the study consisted of 300 patients, and the sample size calculation was performed using the simple random sampling method. With a 95% confidence level and a 5% margin of error, the minimum sample size was calculated to be 165 and the study was conducted with the participation of 184 patients.

Total 184 patients diagnosed T2DM, aged 18 and older, who showed readiness to take part and maintained a communicative mental state, were provided with both verbal and written details about the study before giving informed consent. Patients with shoulder pathology before the diabetes diagnosis, shoulder trauma, acute (presence of injury, infection, fibromyalgia, etc.) and chronic (rheumatoid arthritis, heart and lung diseases, neck hernias, cancers, etc.) diseases causing shoulder pain, and patients with neurological sequelae were not included in the study.

Participants completed a questionnaire covering sociodemographic characteristics, diabetes duration, medication use, and lifestyle factors such as smoking and alcohol consumption. The University of California, Los Angeles (UCLA) Shoulder Score (USS) was administered through face-to-face interviews, all conducted by the same researcher. Shoulder pain was assessed based on patient self-report using the USS, but no imaging or clinical diagnosis was made to determine the underlying pathology.

Diabetes-related complications were identified using ICD-10 codes and clinical records. Diabetic retinopathy (H36.0, E11.3) was confirmed by ophthalmologists through fundus examination or retinal photography. Diabetic neuropathy (G63.2) was diagnosed by neurologists or endocrinologists based on clinical findings and, when necessary, electromyography (EMG). Diabetic nephropathy (N08.3) was confirmed by nephrologists or internists using laboratory results, including albuminuria, serum creatinine, and estimated glomerular filtration rate (eGFR). All data were recorded in the electronic health record system using standardized methods. HbA1c was measured using the capillary zone electrophoresis method (Sebia Capillarys System, Sebia, Norcross, CA). Thyroid dysfunction was evaluated based on serum TSH, fT3, and fT4 levels. Overt hypo- or hyperthyroidism was defined by abnormal TSH and thyroid hormone values. Subclinical hypothyroidism was defined as elevated TSH with normal fT4, and subclinical hyperthyroidism as low TSH with normal fT3 and fT4. Goitre was not considered dysfunction unless hormone levels were also abnormal.^14^ Body mass index (BMI) was calculated as weight (kg) divided by height squared (m²). According to WHO criteria, BMI 18.5–24.99 was normal, 25.0–29.99 pre-obese, and ≥30.0 obese.^15^

The UCLA Shoulder Score (USS), first introduced by Ellman in 1986, is a widely utilized assessment tool.^16^ A Turkish adaptation study validating its reliability was conducted by Büyükdoğan et al. in 2021.^17^ The USS evaluates five key aspects: pain, function, patient satisfaction, flexion strength, and flexion angle, with a total possible score of 35. Pain and function are rated on a scale from 1 to 10, while patient satisfaction, flexor muscle strength, and active flexion angle are scored between 0 and 5. The final score is determined by summing the individual component scores. Based on the scoring system, a total of 34-35 points indicates excellent function, 28-33 points signify good function, 21-27 points indicate moderate function, and 0-20 points represent poor function.^16^

### Statistical Analysis:

The normality of continuous variables (age, USS, HbA1c, diabetes duration, and BMI) was assessed using graphical methods and the Shapiro-Wilks test. All variables except age showed non-normal distribution; thus, medians and interquartile ranges (IQR) were reported. Group comparisons of USS were performed using the Mann–Whitney U test and Kruskal–Wallis analysis, while correlations were evaluated with Spearman’s coefficient. USS scores <28 were classified as poor, and ≥28 as good. Risk factors for poor USS were analyzed using multivariate logistic regression, with results presented as odds ratios (Exp(B)) and 95% confidence intervals. Statistical analysis was conducted using IBM SPSS 21.0 and MS Excel 2007, with p<0.05 considered significant.

## RESULTS

Among the 184 participants, 63 (34.2%) had a poor USS (score <28), while 121 (65.8%) had a good USS (score ≥28). The median USS score was 33.0 (IQR 12.0). Statistically significant negative correlations were found between USS and HbA1c (rho=-0.186, p=0.011), diabetes duration (rho=-0.241, p=0.001), and BMI (rho=-0.232, p=0.002), but no significant correlation with age. The descriptive statistics and relationships between USS and variables are shown in Table-I.

**Table-I:** Relationship between UCLA Shoulder Score and variables.

	USS		
	Median (IQR)	rho	p
Age(years)	59.5 (17.0)	0.138	0.061
Height (cm)	165.0 (14.0)	0.271	<0.001
Weight (kg)	78.0 (21.0)	-0.087	0.240
Hba1c(%)	7.4 (2.4)	-0.186	0.011
Duration of Diabetes (years)	14.5 (14.0)	-0.241	0.001
BMI (kg/m^2^)	28.4 (7.8)	-0.232	0.002

rho: Spearman’s Correlation Coefficient, ***Abbreviations***: IQR: Interquartile Range, BMI:Body Mass Index, USS: UCLA Shoulder Score, HbA1c: Hemoglobin A1c.

Comparison of participants’ demographic characteristics and USS across independent variable groups is presented in Table II. USS scores differed significantly depending on the presence of diabetic retinopathy. For those with diabetic retinopathy, the median (IQR) score was 22.0 (14.0), whereas for those without, it was 33.0 (10.0) (z=4.020, p<0.001). Additionally, a statistically significant difference was observed in the USS of participants based on the presence of hypertension (z=1.999, p=0.046) and thyroid dysfunction (z=2.055, p=0.040) (Table-III).

**Table-II:** Comparison of demographic characteristics and UCLA Shoulder Score of participants in independent variable groups.

		Demographic Characteristics	Test Statistics*		
		n(%)	USS Median (IQR)	Z; χ^2^	p
Gender	Male	87 (47.3)	35.0 (4.0)	z=5.287	<0.001
	Female	97 (52.7)	28.0 (14.0)		
Education Level	Below High School	122(66.3)	31.0(13.0)	z=3.180	0.001
	High School and Above	62(33.7)	35.0(5.0)		
Diabetes Duration	Less Than 10 years	59(32.0)	35.0(6.0)	z=25.31	0.011
	10 years and Above	125(68.0)	33.0(13.0)		
Occupation	Unemployed	77 (41.8) ^b,c,d^	25.0 (13.0)	χ^2^=32.032	<0.001
	Worker	12 (6.5)^a^	35.0 (12.0)		
	Official+Engineer	15 (8.2)^b^	35.0 (3.0)		
	Retired	43 (23.4)^c^	35.0 (4.0)		
	Self Employed	37 (20.1)^d^	33.0 (8.0)		
Antidiabetic Medication	Oral Antidiabetic	96 (52.2)	33.0 (12.0)	χ^2^=1.335	0.721
	Insulin	15 (8.2)	35.0 (11.0)		
	Oral Antidiabetic+Insulin	72 (39.1)	33.0 (13.0)		
Smoking	Yes	51 (27.7)	31.0 (12.0)	χ^2^=4.907	0.086
	No	74 (40.2)	31.0 (13.0)		
	Quit	59 (32.1)	35.0 (6.0)		
Alcohol Consumption	Yes	23 (12.5)	35.0 (3.0)	χ^2^=11.564	0.003
	No	143 (77.7)	31.0 (13.0)		
	Quit	18 (9.8)	35.0 (2.0)		
Night Pain in Shoulder	Yes	91 (49.5)	24.0 (11.0)	z=8.620	<0.001
	No	93 (50.5)	35.0 (2.0)		
Restriction of Shoulder Movement	Yes	77 (41.8)	23.0 (11.0)	z=8.054	<0.001
	No	107 (58.2)	35.0 (2.0)		
BMI Classification	Normal	44 (23.9)^a^	35.0 (13.0)	χ^2^=8.887	0.012
	Pre-obese	73 (39.7)^b^	33.0 (8.0)		
	Obese	67 (36.4) ^a,b^	30.0 (13.0)		

z: Mann Whitney U Test Statistics, χ^2^=Kruskal Wallis Test Statistics, Abbreviations: IQR: Interquartile Range, BMI: Body Mass Index, USS: UCLA Shoulder Score, abcd: Same letters express significant difference between groups.

**Table-III:** Comparison of diabetes complications, other diseases and UCLA Shoulder Score of participants in independent variable groups.

		Demographic Characteristics	Test Statistics*		
		n(%)	USS Median (IQR)	Z	p
Diabetic Retinopathy	No	151 (82.1)	33.0 (10.0)	z=4.020	<0.001
	Yes	33 (17.9)	22.0 (14.0)
Diabetic Nephropathy	No	162 (88.0)	33.0 (11.0)	z=1.904	0.057
	Yes	22 (12.0)	26.0 (15.0)
Diabetic Neuropathy	No	174 (94.6)	33.0 (11.0)	z=1.429	0.153
	Yes	10 (5.4)	22.0 (16.0)
Hypertension	No	73 (39.7)	35.0 (10.0)	z=1.999	0.046
	Yes	111 (60.3)	33.0 (13.0)
Cardiovascular Disease	No	141 (76.6)	33.0 (12.0)	z=0.790	0.430
	Yes	43 (23.4)	33.0 (13.0)
Hyperlipidemia	No	114 (62.0)	33.5 (11.0)	z=1.666	0.096
	Yes	70 (38.0)	31.0 (13.0)
Thyroid Dysfunction	No	151 (82.1)	33.0 (11.0)	z=2.055	0.040
	Yes	33 (17.9)	31.0 (11.0)

z: Mann Whitney U Test Statistics, Abbreviations: USS: UCLA Shoulder Score, IQR: Interquartile Range.

Potential risk factors associated with poor USS in multivariate logistic regression model are shown at Table- IV. The model’s explanatory power was evaluated using Cox & Snell or Nagelkerke R^2^ values. The Nagelkerke R^2^ value of 0.503 in the logistic regression model indicates that the multivariate model explains the response variable (poor USS) to a good extent.

**Table-IV:** Potential risk factors associated with poor UCLA Shoulder Score in multivariate logistic regression model.

Variables	Β	Standard Error	Wald	P	Exp(B)	95% Confidence Interval for Exp(B)	
						Lower	Upper
Constant	-1.227	2.004	0.375	0.540	0.293	-	-
Age (years)	-0.046	0.022	4.277	0.039	0.955	0.915	0.998
Gender (Female)	1.072	0.523	4.192	0.041	2.921	1.047	8.149
BMI (kg/m^2^)	0.021	0.036	0.325	0.568	1.021	0.951	1.096
HbA1c(%)	-0.040	0.104	0.147	0.702	0.961	0.785	1.177
Occupation (Working)	0.023	0.576	0.002	0.968	1.024	0.331	3.164
Retinopathy	1.091	0.546	4.000	0.046	2.978	1.022	8.679
Nephropathy	0.046	0.653	0.005	0.944	1.047	0.291	3.767
Neuropathy	1.001	0.907	1.217	0.270	2.721	0.460	16.102
Duration of Diabetes(years)	0.031	0.027	1.311	0.252	1.031	0.978	1.088
Shoulder Movement Restriction	1.433	0.454	9.982	0.002	4.191	1.723	10.196
Night Shoulder Pain	1.507	0.487	9.577	0.002	4.515	1.738	11.730

***Abbreviations:*** HbA1c: Hemoglobin A1c, BMI: Body Mass Index.

## DISCUSSION

Our study revealed that approximately one in three diabetic patients experienced shoulder pain or dysfunction. By comprehensively analyzing multiple interrelated parameters, we identified several noteworthy associations. Nocturnal shoulder pain was associated with a 4.5-fold increase in risk, while the presence of retinopathy increased this risk by 2.98 times. A positive correlation was observed between higher BMI and greater shoulder pain and dysfunction. Female participants exhibited a 2.92-fold greater likelihood of experiencing these issues compared to males. Moreover, prolonged diabetes duration, particularly beyond 10 years, was associated with a heightened prevalence of shoulder complications. Furthermore, each additional year of age increased the probability of shoulder pain and dysfunction by 4%.

Evidence linking chronic microvascular complications of diabetes to shoulder pain and dysfunction is inconsistent. Cagliero et al. reported higher rates of retinopathy, nephropathy, and neuropathy in patients with shoulder and hand syndromes.^18^ Ramchurn et al. highlighted a connection between DM’s locomotor manifestations and other complications, including nephropathy.^19^ Yian et al. reported that diabetic patients with frozen shoulder complaints had a higher incidence of retinopathy, nephropathy, and neuropathy.^20^ Laslett et al. observed a higher rate of shoulder disability in individuals who underwent eye laser surgery for retinopathy.^21^ Balcı et al. found a significant relationship between AC and retinopathy, but no significant relationship with nephropathy or neuropathy.^22^ Majjad et al. identified a statistically significant relationship between nephropathy and osteoarthritis.^23^ In contrast, Schulte et al. found no significant relationship between passive joint range of motion and retinopathy, nephropathy, or neuropathy.^24^

Our study found a significant inverse relationship between USS and retinopathy, with lower USS scores in patients with DM-related retinopathy. While USS was also reduced in those with DM-related nephropathy or neuropathy, these associations were not statistically significant. Our findings align with those of Laslett and Balcı regarding the link between DM’s microvascular complications and shoulder pain and function.^21^,^22^ Both diabetic retinopathy^25^ and shoulder pathologies in diabetes^9^ may involve tissue damage caused by AGEs, which could explain the higher prevalence of shoulder pathologies in individuals with diabetic retinopathy.

There are discrepancies in the literature regarding the relationship between HbA1c levels and the prevalence of shoulder pathologies. Although some studies have failed to establish a clear association between HbA1c levels and shoulder dysfunction (e.g., Czelusniak et al.^26^), others, including Aydeniz et al.^27^ and Ramchurn et al.^19^, have reported a significant link. These studies suggest that poor glycemic control may contribute to connective tissue degeneration or inflammation. Consistent with these findings, our study demonstrated a statistically significant inverse relationship between HbA1c levels and USS, indicating that elevated HbA1c may play a role in shoulder dysfunction among T2DM patients.

In the study by Majjad et al. investigating MS disorders in DM patients, it was shown that being over 50 years old increases the prevalence of one or more MS disorders.^23^ Balci et al. also found in their study that AC is associated with age.^22^ The study by Chen & Chen suggests that while age may be an important factor, the relationship with the duration of diabetes and AC is stronger.^28^ In a study by Cagliero et al. investigating MS disorders in the hand and shoulder of DM patients, they found a statistically significant association with the duration of diabetes, but not with age.^18^ In our study, the USS score decreased as the duration of diabetes increased and age negatively predicted the USS score in the model created with age. Our study is consistent with the studies by Majjad and Balci in showing an association between age and USS. An increase in age and diabetes duration may be associated with microcirculation disorders or the non-enzymatic glycosylation process, which could adversely affect shoulder pain and function.^29^

Rechardt et al. identified a significant association between shoulder pain and weight-related factors, including BMI, waist circumference, and waist-hip ratio, with the latter two demonstrating the strongest correlations.^30^ Similarly, Cole et al. reported that 37.1% of individuals experiencing shoulder pain or stiffness were classified as obese. Notably, in non-obese participants, diabetes did not significantly influence the prevalence of shoulder symptoms. However, their findings underscored obesity as a more critical determinant of shoulder pain and stiffness than dysregulated glucose metabolism.^5^ Ozkuk and Ates further suggested a potential link between obesity and shoulder pain.^31^ In contrast, Laslett et al. reported no significant difference in BMI between diabetic individuals with and without shoulder pain or functional impairment.^21^

Our findings align with those of Cole and Rechardt, demonstrating a significant relationship between BMI and USS scores.^5^,^30^ Obesity is known to promote the release of pro-inflammatory cytokines such as IL-1, IL-6, and TNF-α,^30^ which have also been implicated in the pathophysiology of shoulder disorders. From this perspective, elevated circulating levels of these cytokines may contribute to shoulder complaints by heightening pain sensitivity or perpetuating chronic inflammation.^30^

The literature indicates a higher prevalence of shoulder pain and dysfunction in females. Laslett et al. investigated shoulder pain and range of motion in diabetic patients and found that females experienced a higher prevalence of both pain and limited mobility.^21^ Similarly, Cagliero et al. reported that shoulder pathologies were more common in diabetic females.^18^ In our study, the total USS score was significantly lower in females. AC, a common cause of shoulder pain and dysfunction, occurs more frequently in females.^32^ This increased prevalence of AC among females may explain the higher rates of shoulder pain and dysfunction observed in our study. Another possible explanation for lower shoulder scores in female participants is that women generally have lower upper-body muscle mass and strength, which is attributed to sex-related differences in skeletal muscle development.^33^ Furthermore, estrogen and other sex hormones may alter the biomechanical characteristics of connective tissues and joint stability, which could play a role in increased sensitivity to pain and poorer shoulder function.^34^ Additionally, among the 77 unemployed participants, 75 were female, and the median USS score was lower in unemployed participants. This could be attributed to the higher prevalence of shoulder pathologies in diabetic women and their greater susceptibility to shoulder musculoskeletal disorders.

This study adds to the literature by highlighting the high prevalence of shoulder dysfunction in patients with T2DM and its associations with retinopathy, female sex, and obesity. Its strength lies in the comprehensive evaluation of multiple risk factors, including microvascular complications, glycemic control, and sociodemographic variables. The findings emphasize the clinical relevance of routine musculoskeletal assessment in diabetic care. Future multicenter and longitudinal studies are needed to clarify causal mechanisms and improve management strategies.

### Limitations

It includes a single HbA1c value and the lack of long-term HbA1c monitoring can be considered as a limitation. Another limitation of the study is that patients assessed for shoulder pain and function were not examined using additional imaging methods, such as computed tomography, magnetic resonance imaging, or ultrasonography of the shoulder joints. A further limitation of the study is the lack of standardized and quantitative pain assessment tools, such as the Visual Analog Scale (VAS) or algometry. Future research should include these methods to allow for more objective and comparable assessment of shoulder pain, independent of functional scoring systems.

## CONCLUSION

In this study, 34.2% of patients with Type-2 diabetes were found to have poor shoulder function, as indicated by a low UCLA Shoulder Score. Retinopathy and female sex were independently associated with 2.98- and 2.92-fold increased odds of shoulder pathology and functional impairment, respectively. These findings underscore the importance of routinely assessing shoulder pain and mobility in diabetic patients, particularly those with diabetic retinopathy, also with obesity, or long-standing diabetes. Clinical attention should also be directed toward female patients and individuals with lower socioeconomic status, as they may be at greater risk of musculoskeletal complications. Routine screening and timely referral to appropriate specialties may help prevent functional impairment and improve quality of life. Enhancing physician awareness regarding the musculoskeletal manifestations of diabetes is essential for comprehensive patient care. Future research should consider adopting a multisystemic approach to diabetes management, incorporating musculoskeletal assessment as a standard component of care. Moreover, multicenter studies are warranted to further explore the etiological factors contributing to shoulder pathologies in diabetic populations, which could improve the generalizability of findings and inform targeted interventions.

### Author’s Contribution:

***ET & AD:*** Concept, design, data acquisition, statistical analysis, methodology development, resource provision, supervision, drafting and editing of the manuscript. Responsible for the integrity of the research. All authors have approved the final version of the manuscript.
